# QTL mapping and identification of linked markers for anthracnose fruit rot resistance in *Capsicum baccatum* L.

**DOI:** 10.3389/fpls.2026.1809315

**Published:** 2026-09-23

**Authors:** Karishma Pasupula, Vanlalneihi Bawitlung, Krishna Sai Karnatam, Sriram Subbaraman, Anand Kumar reddy Chinthaparth, Manoj Balenahalli Parameshwaraiah, Mahebub Shaik, Sandeep Kumar Gullahalli Manjegowda, Lakshmana Reddy Dhoranapalli Chinnappareddy, Naresh Ponnam, Madhavi Reddy Kambham

**Affiliations:** 1 Division of Vegetable Crops, Indian Council of Agricultural Research (ICAR)–Indian Institute of Horticultural Research, Bengaluru, Karnataka, India; 2 School of Agricultural Biotechnology, Punjab Agricultural University, Ludhiana, Punjab, India; 3 Department of Biology, Gus R. Douglass Institute, West Virginia State University, Institute, WV, United States; 4 Division of Crop Protection, Indian Council of Agricultural Research (ICAR)–Indian Institute of Horticultural Research, Bengaluru, Karnataka, India; 5 Division of Basic Sciences, Indian Council of Agricultural Research (ICAR)–Indian Institute of Horticultural Research, Bengaluru, Karnataka, India

**Keywords:** epistasis, genotyping-by-sequencing, marker-assisted selection, quantitative trait loci, resistance, simple sequence repeat markers

## Abstract

Anthracnose fruit rot caused by *Colletotrichum truncatum* is a major constraint in Chilli production, leading to heavy pre- and post-harvest losses. Developing resistant cultivars through marker-assisted breeding is the most sustainable management strategy. Identification of molecular markers tightly linked to anthracnose fruit rot resistance loci in *Capsicum baccatum* accessions will be of great use in chili crop improvement programs to introgress resistance loci into elite backgrounds. Inheritance studies in an F₂ population (IIHR 4491 × PBC81) indicated inhibitory gene action (13S:3R) governing resistance against *C. truncatum* isolate IIHR Ct-1. Genotyping-by-sequencing identified 8,644 high-quality single nucleotide polymorphism (SNP) markers distributed across all chromosomes, ranging from 533 on chromosome 5 to 1,130 on chromosome 3. A genome-wide association study identified 19 SNPs significantly associated with resistance mainly on chromosomes 2 and 6. The two quantitative trait locus (QTL) regions were further refined to 836.18 kb on chromosome 2 (*AnR-PBC81-C2*) and 135.15 kb on chromosome 6 (*AnR-PBC81-C6*) through target loci-based QTL mapping. Epistasis analyses indicated significant epistatic interactions between these two QTL hotspots. Seventeen candidate genes involved in plant defense mechanisms were predicted within these regions. The identified markers were also validated in another F₂ population (IHR4491 × PBC80). Meta-QTL analysis identified 36 MQTLs, with *AnR-PBC81-C2* assigned to MQTL2.2. The QTL on chromosome 6 (*AnR-PBC81-C6*) did not colocalize with any previously reported QTLs, indicating that it represents a novel resistance locus. The refined QTLs and associated developed SSR markers can be utilized to accelerate marker-assisted introgression breeding for anthracnose fruit rot disease resistance.

## Introduction

Chilli (*Capsicum* spp.) is an important vegetable-cum-spice crop cultivated extensively across tropical and subtropical regions of the world, valued for its culinary, nutritional, cosmeceutical, and pharmaceutical applications. India is one of the leading producers of chilli, with an annual production of approximately 5.754 million metric tons (MT) of green chilli and 2.464 million MT of dry chilli (NHB, 2025–2026). Chilli contributes significantly to the Indian economy, accounting for approximately 39.8% of the total spice exports, with earnings of USD 1.37 billion during 2023–2024 (Spices Board of India, 2024). Chilli cultivation is threatened by several biotic and abiotic stresses, among which anthracnose fruit rot is one of the most devastating. This disease, caused by a complex of *Colletotrichum* species, primarily *C. truncatum*, *C. scovillei*, and *C. siamense*, affects fruits during both pre- and post-harvest stages, leading to unmarketable produce and substantial yield losses. Anthracnose fruit rot is characterized by sunken necrotic lesions with concentric rings and spore masses, causing yield losses ranging from 15% to 80% (Montri et al., 2009; Ridzuan et al., 2018). The disease spreads rapidly through rain-splash or wind-borne conidia, and its management through fungicide application is constrained by residue concerns, pathogen variability, and resistance development (Than et al., 2008; Mongkolporn and Taylor, 2018). Deployment of host plant resistance is the most sustainable management strategy. However, most cultivated *Capsicum annuum* genotypes are highly susceptible to *Colletotrichum* spp., necessitating the identification and introgression of resistance genes from related species. Resistant sources have been reported mainly in *C. baccatum* (PBC80, PBC81, and PI594137) and *C. chinense* (PBC932) (AVRDC, 1998; Gniffke et al., 2013). Nonetheless, practical breeding applications are limited by cross-incompatibility between *C. annuum* and *C. baccatum*, primarily due to pre- and post-zygotic barriers such as embryo abortion, hybrid sterility, and segregation distortion (Voorrips et al., 2004; Yoon and Park, 2005). Inheritance studies have revealed diverse genetic models, including monogenic recessive (Pakdeevaraporn et al., 2005; Kim et al., 2008; Rahman and Akanda, 2022), monogenic dominant (Mahasuk et al., 2009), duplicate gene action (Mahasuk et al., 2013; Kethom and Mongkolporn, 2021), and polygenic quantitative control (Lee et al., 2010; Giacomin et al., 2020). These complexities have limited the precision of conventional breeding in developing resistant hybrids or varieties.

Recent advances in next-generation sequencing (NGS) tools, particularly genotyping-by-sequencing (GBS), have facilitated high-resolution genome-wide single nucleotide polymorphism (SNP) discovery and quantitative trait locus (QTL) mapping (Elshire et al., 2011). Integration of GBS with SSR and SNP-based marker systems enhances genetic resolution and accelerates marker-assisted selection (MAS) (Han et al., 2018; Siddique et al., 2019; Lee et al., 2016). In *Capsicum*, GBS-based linkage maps have facilitated the identification of resistance loci against diverse pathogens (Kim et al., 2017; Kumar et al., 2022). Significant progress has been made in identifying QTLs for anthracnose fruit rot disease resistance, notably in the resistant sources PBC80 and PBC932. Mahasuk et al. (2016) mapped major and minor QTLs in the F₂ population of *C. chinense* “PBC932” and *C. baccatum* “PBC80” against *C. truncatum* isolate 158ci and *C. scovillei* pathotypes (PCa2 and PCa3). Nanda et al. (2016) identified SSR markers linked to resistance in an F₂ population derived from PBC80 × SB1 against *C. capsici* isolate Cc38. A major QTL, *AnRGO5*, was fine-mapped on chromosome 5 in backcross populations of “PBC932” × *C. annuum* “77013” against *C. acutatum* using KASP and InDel markers (Zhao et al., 2020). Stage-specific major and minor QTLs mainly on chromosomes 3, 4, and 8 were identified against *C. scovillei* isolate MJ5 in 97 recombinant inbred lines (RILs) of PBC80 × CA1316 and were mapped (Kethom and Mongkolporn, 2021). As evidenced by different studies reporting QTLs on varied chromosomes, it is very clear that resistance mechanism varies with the species of *Colletotrichum* and strain. Therefore, identifying the genomic regions and associated markers conferring resistance against prevalent region-specific virulent strains and exploring them in marker-assisted breeding is the best advocated strategy. Limited information is available on QTLs and tightly linked markers associated with resistance against *Colletotrichum* isolates of India. Moreover, integration of QTL information across studies through meta-QTL (MQTL) analysis enables the identification of stable consensus genomic regions with reduced confidence intervals, thereby improving the broad-spectrum resistance breeding. Therefore, the present study was undertaken to identify genomic regions associated with resistance against anthracnose fruit rot caused by prevalent virulent *C. truncatum* isolate IIHR Ct-1 in *C. baccatum* using GBS-derived SNPs, further refine the QTL hotspots through mining of SSR markers in the targeted loci and integrate the findings with MQTL analysis for identifying stable resistance hotspots, and identify markers associated with resistance for MAS to enable accelerated breeding.

## Materials and methods

### Plant materials and phenotypic evaluation for anthracnose fruit rot resistance

A biparental mapping population was developed by crossing the highly susceptible accession IIHR 4491 with the highly resistant accession PBC 81. F_1_ was selfed to produce F_2_ segregating population (Vanlalneihi et al., 2023). To validate the identified SSR markers associated with QTLs on chromosomes 2 and 6 for anthracnose fruit rot resistance, an alternate F₂ mapping population consisting of 233 individuals was developed from a cross between IIHR 4491 (susceptible) and PBC 80 (resistant).

Phenotypic screening was carried out under controlled laboratory conditions using red-ripe fruits from each individual F₂ plant (five fruits per plant). Artificial inoculation was performed with *C. truncatum* (IIHR Ct-1 isolate; GenBank accession no. MN873012) using the pin-pricking method and inoculating with a spore suspension of 5 × 10⁵ conidia/mL. Each fruit was inoculated with 10 µL of inoculum, and disease development was evaluated at 14 days post-inoculation (dpi) (Vanlalneihi et al., 2023; Yoon, 2003). Resistance was quantified based on lesion length (LL), lesion width (LW), lesion area (LA), percent lesion area (PLA), and disease severity score (DS) (Pakdeevaraporn et al., 2005). Fruits were categorized into six disease reaction classes: highly resistant (HR; score 0), resistant (R; score 1), moderately resistant (MR; score 3), moderately susceptible (MS; score 5), susceptible (S; score 7), and highly susceptible (HS; score 9).

### Genotyping-by-Sequencing

A total of 357 F₂ plants of IIHR 4491 × PBC81 cross along with their parental lines were subjected to GBS using the DArTSeq™ platform (Diversity Arrays Technology, Canberra, Australia). Genomic DNA of leaf tissues was extracted using the cetyltrimethylammonium bromide method as described by Doyle and Doyle (1987). Sequencing was performed at Diversity Arrays Technologies, Canberra, Australia, through proprietary DArTSeq™ technology (Sansaloni et al., 2011). Genome complexity was reduced using the restriction enzymes *PstI* and *MseI*, and digestion–ligation procedures were performed as per the standard methods reported by Kilian et al. (2012). Barcodes as described were used (Elshire et al., 2011). For polymerase chain reaction (PCR) selective amplification of fragments with both cutting ends of *PstI* and *MseI*, selective primer pairs for the *PstI* adapter and the *MseI* adapter were used. Polymerase chain reaction PCR conditions were 1 min at 94 °C for initial denaturation; 30 cycles each consisting of 20 s at 94 °C for denaturation, 30 s at 58 °C for annealing, and 45 s at 72 °C for extension; and a final extension step for 7 min at 72 °C. After PCR, bulking of amplification products in equimolar amounts from each sample was carried out and applied to c-Bot (Illumina) bridge PCR followed by sequencing on Illumina Hiseq 2500 (Ren et al., 2015). Sequence data were processed using the proprietary DArT analytical pipelines (Ren et al., 2015), and the reads were aligned to the *Capsicum baccatum* reference genome (GenBank Accession: CM008444.1). SNP filtering criteria of call rate > 0.2, Rep Ave > 0.86, Avg Count Snp > 2.5, Avg Count Ref > 2.5, Avg PIC > 0.05, ratio Avg CountRefAvgCountSnp < 5, ratioAvgCountRefAvgCountSnp > 0.2, and FreqHets < 0.8 were used in processing. Sequencing data have been deposited in the NCBI Sequence Read Archive under BioProject accession PRJNA1391877.

### Genome-wide marker–trait association analysis

A GWAS-like algorithm was employed for association analysis between genotypic data (SNPs) and anthracnose fruit rot resistance (LL, LW, and LA), as these are less affected by scoring errors in heterozygous material (DArTSeq pipelines). A linear mixed model (LMM) was used to obtain marker–trait associations. The GWAS analysis was conducted using the GWAS function in the R package *rrBLUP*, which implements the mixed-model association eXpedited (EMMAX) algorithm (Kang et al., 2010). Marker–trait associations were evaluated by calculating the –log₁₀(*p*) value for each SNP. By applying the mixed model, Manhattan plots were generated for each trait. The GWAS marker–trait association analysis was implemented using the KDCompute platform of DArTseq (https://www.kddart.org/kdcompute.html and https://www.diversityarrays.com/services/data-analytics/qtl-association-mapping-gwas/).

### Refinement of genomic regions through *in silico* microsatellite marker mining

For refinement of genomic loci associated with anthracnose fruit rot resistance, *in silico* mining of simple sequence repeats (SSRs) was performed within the identified targeted genomic regions (QTL intervals on chromosome 2 and chromosome 6) using the *C. baccatum* reference genome (GenBank Accession: CM008444.1) (Das et al., 2021) through Krait software (Du et al., 2018). SSR mining was performed on broader genomic intervals spanning 126.60–142.20 Mb (~15.60 Mb) on chromosome 2 and 247.00–252.30 Mb (~5.30 Mb) on chromosome 6. The search parameters were optimized to detect mono- to hexa-nucleotide repeat motifs, with minimum thresholds of ≥20 for mono-, ≥9–12 for di-, ≥6–10 for tri-, and ≥5–8 for tetra-, penta-, and hexa-nucleotide repeats. Motif identification followed Level 3 complementary motif standardization. Primer pairs were designed from 100-bp flanking regions with primer design parameters including a sequence length of 18–22 bp (optimum 20 bp), a GC content of 40–60%, a GC clamp of 2, and melting temperatures between 55 and 60 °C with a maximum allowable *T*_m_ difference of 3 °C. Primer specificity was ensured by applying filters for poly-X regions (maximum length of 4 bp), self-complementarity, hairpin Δ*G* (≤47.0), and end stability (≤100) (Portis et al., 2007).

### Validation of available molecular markers associated with Anthracnose fruit rot resistance

In addition to SSR markers designed in the targeted loci, 50 markers associated with anthracnose fruit rot resistance from earlier studies were used in genotyping. These markers were used as reference loci for initial polymorphism assays and marker–trait association studies (**Supplementary Table 1**). PCR amplification protocol was optimized. Each 16-µL PCR reaction contained 3.0 µL of template DNA (100–120 ng), 2.0 µL each of forward and reverse primers (5 pmol, final concentration 0.625 µM), 2.0 µL of dNTP mix (1 mM, final concentration 0.125 mM each), 2.0 µL of 10× Taq buffer, 0.5 µL of Taq DNA polymerase (1 U; 3B BlackBio Biotech India Ltd.), and 4.5 µL of nuclease-free Milli-Q water. PCR amplification of SSR markers was performed using an Eppendorf thermal cycler following a touchdown protocol to enhance primer specificity. The thermal cycling conditions were an initial denaturation at 94 °C for 5 min, followed by 10 cycles consisting of denaturation at 94 °C for 45 s, annealing starting at 60 °C with a decrement of 1 °C per cycle for 30 s, and extension at 72 °C for 1 min. This was followed by 25 cycles of denaturation at 94 °C for 45 s, annealing at 55 °C for 30 s, and extension at 72 °C for 1 min. A final extension step was carried out at 72 °C for 6 min. All parental polymorphic markers were genotyped in 230 F₂ individual plants of IHR 4491 (susceptible) and PBC 81 (resistant) cross. PCR-amplified products were resolved on 4% agarose gels or 6% PAGE gels and genotypes were scored using UVtec software and recorded as binary data.

### QTL analysis for main and epistatic effects

Genetic linkage map construction and QTL analysis were conducted using QTL IciMapping v4.2 (Meng et al., 2015). Inclusive Composite Interval Mapping for additive effects (ICIM-ADD) and epistatic effects (ICIM-EPI) were employed using phenotypic data (LL, LW, LA, PLA, and DS) and genotypic marker data. Mapping parameters included a step size of 1.0 cM, deletion of missing phenotypes, and a PIN value of 0.001. A LOD threshold was similarly established via 1,000 permutations (α = 0.05). ICIM-EPI facilitated the identification of significant digenic epistatic interactions contributing to resistance. To further validate epistatic QTLs identified via ICIM-EPI, a Bayesian model-based approach was applied using the R package R/qtl bim (Yandell et al., 2007) for high-resolution detection of interacting loci associated with resistance. Bayesian epistatic QTL mapping was performed using the *qb.scanone()* and *qb.scantwo()* functions implemented in the R/qtlbim package under a model allowing pairwise interactions. The analysis used a burn-in of 10,000 iterations and 50,000 MCMC iterations. Significant QTL–QTL interactions were evaluated using posterior probabilities and Bayes factors, and epistatic effects were visualized via two-dimensional interaction plots based on posterior-derived *p*-values.

### QTL data compilation and meta-QTL analysis

A thorough and systematic literature review was conducted using Google Scholar and PubMed Central, to compile information on QTL studies associated with resistance to major diseases of *Capsicum* spp., including anthracnose fruit rot, *Phytophthora* leaf blight, *Phytophthora* root rot, bacterial wilt, bacterial leaf spot, and Cucumber Mosaic Virus (CMV), reported up to the year 2025 (**Supplementary Table 2**). Relevant studies were identified using combinations of keywords including “Capsicum”, “QTL”, “anthracnose resistance”, “disease resistance”, “*Colletotrichum*”, “*Phytophthora capsici*”, “bacterial wilt”, and “CMV resistance”. A total of 14 studies comprising 119 QTLs were included in the MQTL analysis. Extracted information included (i) genetic position of QTLs, (ii) chromosome location, (iii) LOD scores, (iv) proportion of phenotypic variation explained (*R*² or PVE), (v) type and size of the mapping population used, (vi) peak positions of QTLs, and (vii) flanking markers name (95% confidence interval, CI). In cases where peak positions were not reported, the midpoint between flanking markers was considered as the QTL peak (Karnatam et al., 2023). For studies lacking CI data, the 95% CI was estimated using population-specific formulas: F₂ populations: CI = 530/(PVE × *N*), where *N* is the mapping population size and PVE is the phenotypic variance explained by the QTL (Sharma et al., 2023; Goyal et al., 2024).

A consensus genetic map was developed by integrating genetic maps of Cheng et al. (2016), Zhu et al. (2019); Lee et al. (2016), and the genetic map generated in the present study using the LPmerge package in R (Endelman and Plomion, 2014), following the standard approach of Saini et al. (2022) and Karnatam et al. (2023). QTL projection and MQTL analysis were performed using BioMercator v4.2.3 (Arcade et al., 2004) following the two-step approach of Veyrieras et al. Optimal MQTL models were selected based on Bayesian Information Criterion (BIC), Akaike Information Criterion (AIC), corrected AIC (AICc), AIC3, and Average Weight of Evidence (AWE), and singleton QTLs were excluded. MQTL positions were assigned based on the *C. baccatum* reference genome (GenBank accession: CM008444.1), and MQTL confidence intervals, LOD score, and PVE (%) are provided in **Supplementary Table 12**. MQTLs were named sequentially by chromosome and physical order, and PVE values were calculated as the mean of constituent QTLs (Gudi et al., 2022).

## Results

### Phenotypic variation and inheritance of anthracnose fruit rot resistance

The parental lines exhibited expected contrasting reactions, PBC 81 showed highly resistant response, and IHR 4491 showed highly susceptible reaction to *C. truncatum* isolate IIHR Ct-1. Phenotypic screening of an F₂ population of IHR 4491 × PBC 81 cross revealed significant variation for lesion traits, i.e., LL, LW, LA, PLA, and DS. For inheritance analysis, HR and R classes (score 0–1) were grouped as resistant, whereas MR, MS, S, and HS classes (score 3–9) were considered susceptible. Among the 369 F₂ individuals evaluated, 80 were classified as resistant and 289 were classified as susceptible. Chi-square analysis showed a good fit to the expected 13 susceptible:3 resistant segregation ratio (χ² = 2.16, *p* > 0.05), indicating inhibitory gene action governing anthracnose fruit rot resistance against *C. truncatum* isolate IIHR Ct-1. Furthermore, LA and PLA showed high variance (25.31 and 78.18, respectively), and their distributions deviated from normality (*p* < 0.01, Shapiro–Wilk test), with notable skewness and kurtosis (**Supplementary Table 3**).

### Genome-wide distribution of SNPs and marker–trait association analysis for anthracnose fruit rot resistance

GBS of the F₂ mapping population of IHR 4491× PBC 81 cross yielded a total of 8,644 high-quality SNP markers, distributed across all chromosomes. Marker density ranged from 533 (chromosome 5) to 1,130 (chromosome 3). Chromosome 6 (962 SNPs) and chromosome 4 (826 SNPs) showed dense marker coverage, while other chromosomes had moderate SNP counts varying between 543 on chromosome 2 and 849 on chromosome 8 (**Supplementary Table 4**; **Supplementary Figure 1**). Linkage disequilibrium (LD) decay analysis indicated high LD at short physical distances (mean *r*² ≈ 0.37–0.42 within 0–0.1 Mb), followed by a gradual decline with increasing distance. The *r*² value dropped below the threshold of 0.2 beyond ~2 Mb, suggesting an LD block size of approximately 2 Mb in the mapping population (**Supplementary Table 5**; **Supplementary Figure 2**). To identify genomic regions associated with anthracnose fruit rot resistance, a GWAS-like marker–trait association analysis was performed using an LMM with false discovery rate (FDR) correction at 0.1 in the biparental F₂ population. The analysis identified SNPs significantly associated with resistance-related traits, including LL, LW, and PLA. A total of 19 SNPs were significantly associated with anthracnose fruit rot resistance, exceeding the –log₁₀(*p*) threshold of 2.82 and designated as quantitative trait nucleotides (QTNs) (**Table 1**; **Figure 1**). Key QTNs were concentrated on chromosome 2, at positions 141,379,584 and 141,788,365 bp, showing strong associations with LL and LW. Another significant minor-effect locus was identified on chromosome 6 (242,467,673 and 247,161,384 bp), which showed consistent associations with resistance traits. Overall, two major genomic regions each on chromosome 2 and chromosome 6 and other significant minor QTNs on chromosomes 7 and 12 were detected supporting the quantitative nature of inheritance.

**Table 1:** Significant SNPs associated with anthracnose fruit rot resistance caused by *C. truncatum*.

Candidate SNP	SNP	Chromosome	Physical position (bp)	Linkage group	Genetic position (cM)	−log10(*p*) PLA	−log10(*p*) LL	−log10(*p*) LW
Candidate 1	13:T>G	CBv1.2. scaffold1929	22,007	NA	NA	3.704	2.931	2.972
Candidate 2	11:G>A	Chromosome 2	141,379,584	chr2	67.322	3.298	2.674	3.571
Candidate 3	52:A>G	Chromosome 2	141,788,365	chr2	67.322	3.296	2.658	3.912
Candidate 4	52:C>T	CBv1.2. scaffold 515	1,029,032	NA	NA	3.260	2.396	2.656
Candidate 5	37:C>T	Chromosome 7	188,536,848	chr7	76.480	3.207	2.899	2.906
Candidate 6	57:A>C	Chromosome 7	13,116,521	NA	NA	3.146	2.655	2.812
Candidate 7	5:G>A	NA	NA	chr2	67.322	3.133	2.827	3.658
Candidate 8	21:T>A	Chromosome 2	139,136,803	chr2	65.225	3.035	2.727	3.644
Candidate 9	22:G>A	Chromosome 12	213,032,790	NA	NA	3.035	2.589	3.569
Candidate 10	32:T>C	Chromosome 2	129,826,079	chr2	52.623	3.011	2.153	3.183
Candidate 11	46:C>T	Chromosome 6	247,874,886	NA	NA	2.970	3.096	2.887
Candidate 12	27:T>G	Chromosome 6	250,438,400	NA	NA	2.949	2.655	3.109
Candidate 13	9:G>A	NA	NA	chr6	120.439	2.893	2.819	2.991
Candidate 14	48:C>T	Chromosome 6	242,467,673	chr6	120.254	2.882	3.042	3.097
Candidate 15	62:G>A	Chromosome 6	249,464,485	NA	NA	2.874	3.554	2.794
Candidate 16	38:A>T	Chromosome 6	252,255,814	chr6	122.703	2.871	2.837	2.988
Candidate 17	11:G>A	Chromosome 6	251,408,637	NA	NA	2.867	2.831	2.960
Candidate 18	59:A>G	Chromosome 6	247,161,384	chr6	120.102	2.827	2.739	2.908
Candidate 19	51:G>A	Chromosome 2	126,666,827	NA	NA	2.821	2.482	3.273

Physical position (bp) indicates the genomic coordinates of SNPs based on the *Capsicum baccatum* reference genome (GenBank accession: CM008444.1). Genetic position (cM) represents the marker position on the linkage map. Values shown for percent lesion area (PLA), lesion length (LL), and lesion width (LW) are −log10(*p*) scores obtained from the GWAS-like marker–trait association analysis. NA indicates that the SNP could not be assigned to a linkage group or genetic position.

**Figure 1:**
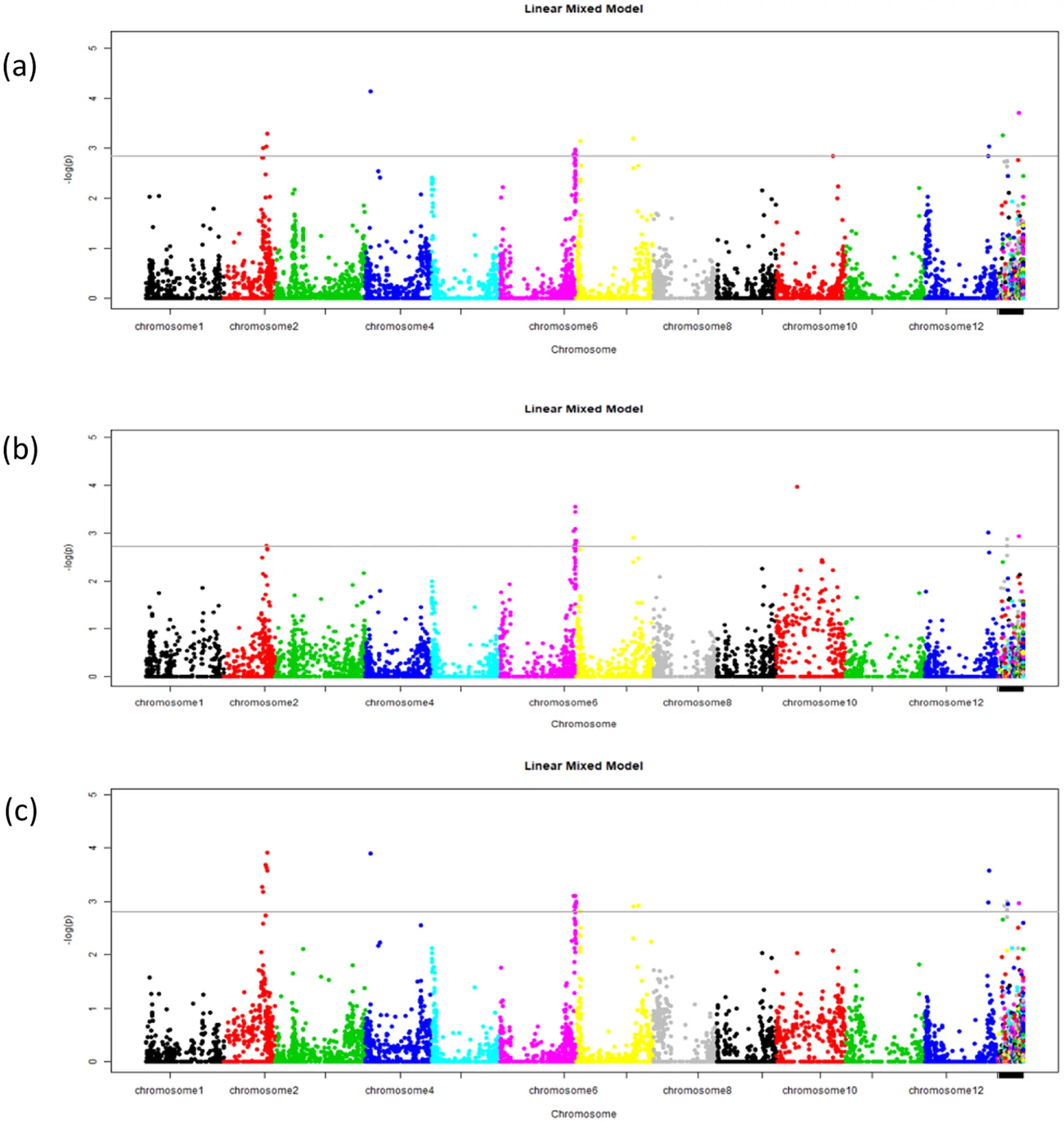
Manhattan plots showing significant SNPs identified through GBS-based marker–trait association analysis for anthracnose fruit rot resistance caused by *C. truncatum* isolate IIHR Ct-1: **(a)** percent lesion area (PLA), **(b)** lesion length (LL), and **(c)** lesion width (LW). The horizontal line indicates the significance threshold [−log10(*p*) = 2.82] used for identifying significant marker–trait associations in the GWAS-like analysis.

### QTL refinement and validation

The two major genomic regions were targeted: 126.60–142.20 Mb (~15.60 Mb) on chromosome 2 and 247.00–252.30 Mb (~5.30 Mb) on chromosome 6 were mined for SSRs, yielding 1,025 perfect SSRs with a combined length of 20,922 bp and an average motif size of 37.77 bp. The valid sequence length was 19.17 Mb (GC content: 34.62%), and SSRs covered 0.11% of the total sequence, with a relative abundance of 28.9 loci/Mb. Among these, di-nucleotide repeats were most frequent (51.4%), followed by tri-nucleotide (33.6%) and mono-nucleotide (8.62%) repeats. Tetra-, penta-, and hexa-nucleotide motifs were rare (<7% combined), with penta-nucleotides showing the longest average repeat length (83.6 bp). Overall, 498 SSR markers (408 markers within QTL on chromosome 2 and 90 within QTL on chromosome 6) were designed for refining the genomic regions on chromosome 2 and chromosome 6 (**Supplementary Figures 1**–**S6**).

A total of 548 SSR markers (498 markers within two QTL regions and 50 markers from published literature) were screened between the resistant parent PBC81 and susceptible parent IHR 4491 to identify parental polymorphic markers. Out of these, 46 markers (8.39%) showed parental polymorphism, i.e., 31 markers within the QTL region on chromosome 2, 12 markers within the QTL region on chromosome 6, and 3 markers from the earlier reported studies of Lee et al. (2011) and Garg et al. (2014) (**Supplementary Table 5**; **Supplementary Figures 7**–**S9**). Lee et al. (2011) reported HpmsE092 linked to resistance against *C. acutatum* and *C. capsici* in PBC 81, while Garg et al. (2014) identified CAMS 351 and CAMS 460 associated with resistance in Punjab Lal. All these parental polymorphic markers were genotyped in 230 F_2_ individual plants in IIHR4491 × PBC81 cross. QTL-specific linkage maps were constructed, which spanned across 474.53 cM on chromosome 2 and 95.08 cM on chromosome 6 (**Supplementary Figures 3**, **S4**). ICIM-ADD analysis refined two QTL regions associated with anthracnose fruit rot resistance. On chromosome 2, a single QTL region spanning 122.0–177.0 cM was consistently associated with LL and LA. Within this region, the peak for LL was mapped at 150.0 cM (CABQ2.62–CABQ2.157; LOD = 3.90, PVE = 7.30%), while LA was associated with peaks at 122.0 cM (LOD = 8.20, PVE = 4.19%) and 177.0 cM (LOD = 6.61, PVE = 3.81%). All QTLs exhibited negative additive effects, confirming the beneficial contribution of resistant parent alleles. On chromosome 6, a minor-effect QTL region flanked by CABQ6.2 and CABQ6.27 was detected, which influenced LA, PLA, and DS with LOD values ranging from 1.09 to 1.65 and PVE of 3.62% (**Table 2**; **Figure 2**; **Supplementary Tables 7**, **S8**). Low *R*² values from ICIM-ADD further implied multiple minor-effect loci for resistance. On chromosome 2, two QTLs associated with LA were detected due to less marker polymorphism, and the extent of LD decay is approximately 2 Mb, so these three QTLs are considered as a single major QTL region designated *AnR-PBC81-C2*, which was refined from 15.60 Mb to 836.18 kb flanked by markers CABQ2.62 and CABQ2.341 (148.44–298.79 cM). On chromosome 6, a minor QTL designated *AnR-PBC81-C6* was refined from 5.30 Mb to 135.15 kb, flanked by CABQ6.2 and CABQ6.78 (29.57–66.09 cM).

**Table 2:** ICIM-ADD for refined QTL intervals for anthracnose fruit rot lesion traits in *Capsicum baccatum*.

QTL	Trait	Chromosome	Genetic position (cM)	Left marker	Right marker	LOD	PVE (%)	Add	Dom	Left CI	Right CI
*AnR-PBC81-C2*	LL	2	150	CABQ2.62	CABQ2.157	3.898	7.30	−0.459	−0.198	130.5	152.5
	LA	2	122	CABQ2.190	CABQ2.62	8.203	4.19	−4.719	−6.405	118.5	128.5
	LA	2	177	CABQ2.157	CABQ2.172	6.611	3.81	−4.280	−5.728	171.5	182.5
*AnR*−*PBC81-C6*	LL	6	45	CABQ6.2	CABQ6.27	1.650	2.98	−0.291	−0.265	23.5	58.5
	LW	6	38	CABQ6.2	CABQ6.27	1.414	3.62	−0.296	−0.181	21.5	62.5
	LA	6	47	CABQ6.2	CABQ6.27	1.086	1.15	−1.081	−0.332	33.5	65.5
	PLA	6	39	CABQ6.2	CABQ6.27	1.257	2.38	−2.069	−2.076	23.5	59.5
	DS	6	41	CABQ6.2	CABQ6.27	1.298	1.99	−0.598	−0.498	20.5	64.5

LL, lesion length; LW, lesion width; LA, lesion area; PLA, percent lesion area (%); DS, disease severity score; LOD, logarithm of odds; PVE, phenotypic variance explained (%); Add, additive effect; Dom, dominance effect; CI, confidence interval.

**Figure 2:**
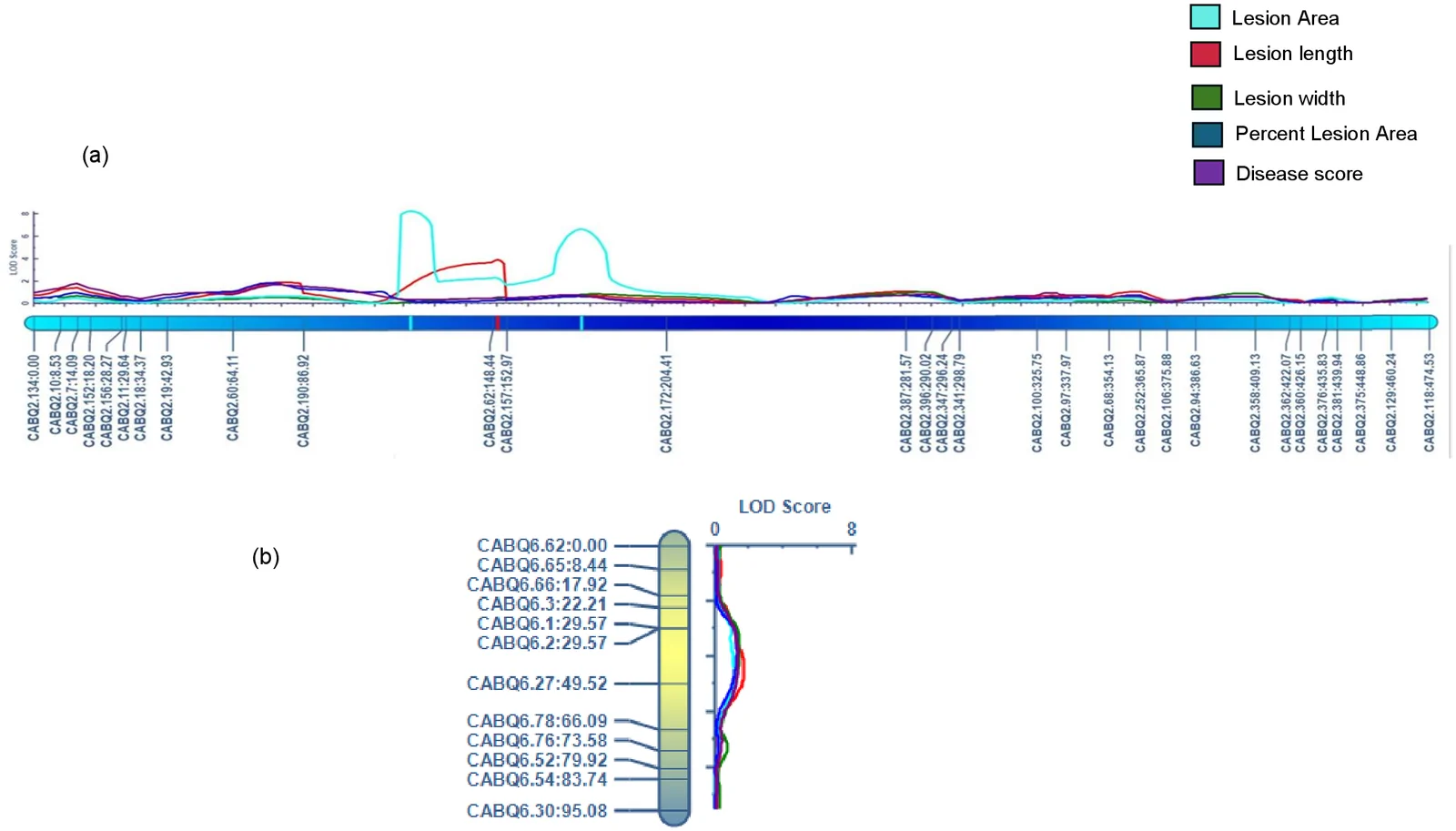
QTL-LOD profiles for anthracnose fruit rot resistance on **(a)** chromosome 2 and **(b)** chromosome 6.

### Epistatic and Bayesian QTL mapping

ICIM-EPI analysis revealed 10 digenic interactions (inter- and intra-chromosomal) contributing to anthracnose fruit rot resistance. Inter-chromosomal interactions were detected for lesion traits, including LA between chromosome 2 (245.0 cM, CABQ2.172–CABQ2.387) and chromosome 6 (55.0 cM, CABQ6.159–CABQ6.78) (LOD = 16.71, PVE = 5.62%), and PLA between chromosome 2 (230.0 cM, CABQ2.172–CABQ2.387) and chromosome 6 (70.0 cM, CABQ6.78–CABQ6.76) (LOD = 10.54, PVE = 6.71%). Intra-chromosomal epistasis was frequent within chromosome 2, with interactions identified for LL (0.0–260.0 cM; LOD = 5.84, PVE = 18.43%), LW (0.0–260.0 cM; LOD = 6.78, PVE = 10.92%), LA (260.0–305.0 cM; LOD = 23.25, PVE = 5.84%), PLA (245.0–415.0 cM; LOD = 15.15, PVE = 7.06%), and DS (190.0–220.0 cM; LOD = 16.24, PVE = 4.33%). Additional interactions for PLA were also observed on chromosome 6 (40.0–70.0 cM interval). These findings indicate the involvement of several other minor genes apart from major inhibitory gene action of disease resistance (**Table 3**; **Figure 3**).

**Table 3:** Epistatic QTLs associated with anthracnose fruit rot lesion traits using ICIM-EPI model-refined QTL intervals.

Trait	Genetic position 1 (cM)	Left marker 1	Right marker 1	Genetic position 2 (cM)	Left marker 2	Right marker 2	LOD	PVE (%)	Add 1	Add 2	Dom 1	Dom 2	Add × Add	Add × Dom	Dom × Add	Dom × Dom
LL	0	CABQ2.134	CABQ2.10	260	CABQ2.172	CABQ2.387	5.841	18.43	0.939	−0.699	0.133	0.572	−0.308	−1.401	1.577	−1.122
LL	50	CABQ6.27	CABQ6.78	70	CABQ6.78	CABQ6.76	6.133	8.91	−1.002	0.707	−1.052	0.078	−0.430	1.492	−1.485	0.477
LW	0	CABQ2.134	CABQ2.10	260	CABQ2.172	CABQ2.387	6.779	10.92	0.760	−0.665	0.480	0.851	−0.188	−1.119	1.619	−1.737
LA	260	CABQ2.172	CABQ2.387	305	CABQ2.341	CABQ2.100	23.248	5.84	−3.733	3.367	3.508	2.916	−2.867	10.447	−5.994	−8.574
LA	40	CABQ6.2	CABQ6.27	45	CABQ6.2	CABQ6.27	12.719	5.40	3.842	−4.515	−4.548	6.412	−3.761	−3.583	4.552	−6.907
LA	245	CABQ2.172	CABQ2.387	55	CABQ6.27	CABQ6.78	16.710	5.62	−1.454	−2.661	−4.667	−4.868	2.369	1.3532	1.905	16.325
PLA	245	CABQ2.172	CABQ2.387	415	CABQ2.358	CABQ2.362	15.154	7.06	−4.410	8.669	−2.530	4.265	−5.257	11.824	−13.830	−9.999
PLA	230	CABQ2.172	CABQ2.387	70	CABQ6.78	CABQ6.76	10.537	6.71	5.559	1.513	−10.491	−1.446	8.470	−13.914	0.449	−0.533
PLA	40	CABQ6.2	CABQ6.27	70	CABQ6.78	CABQ6.76	10.749	4.64	−9.474	7.039	−7.724	3.145	−5.109	17.901	−7.878	−3.295
DS	190	CABQ2.157	CABQ2.172	220	CABQ2.172	CABQ2.387	16.238	4.32	−2.015	2.167	−4.227	3.155	0.162	1.434	−2.158	0.899

LL, lesion length; LW, lesion width; LA, lesion area; PLA, percent lesion area (%); DS, disease severity score; LOD, logarithm of odds; PVE, phenotypic variance explained (%); Add, additive effect; Dom, dominance effect.

**Figure 3:**
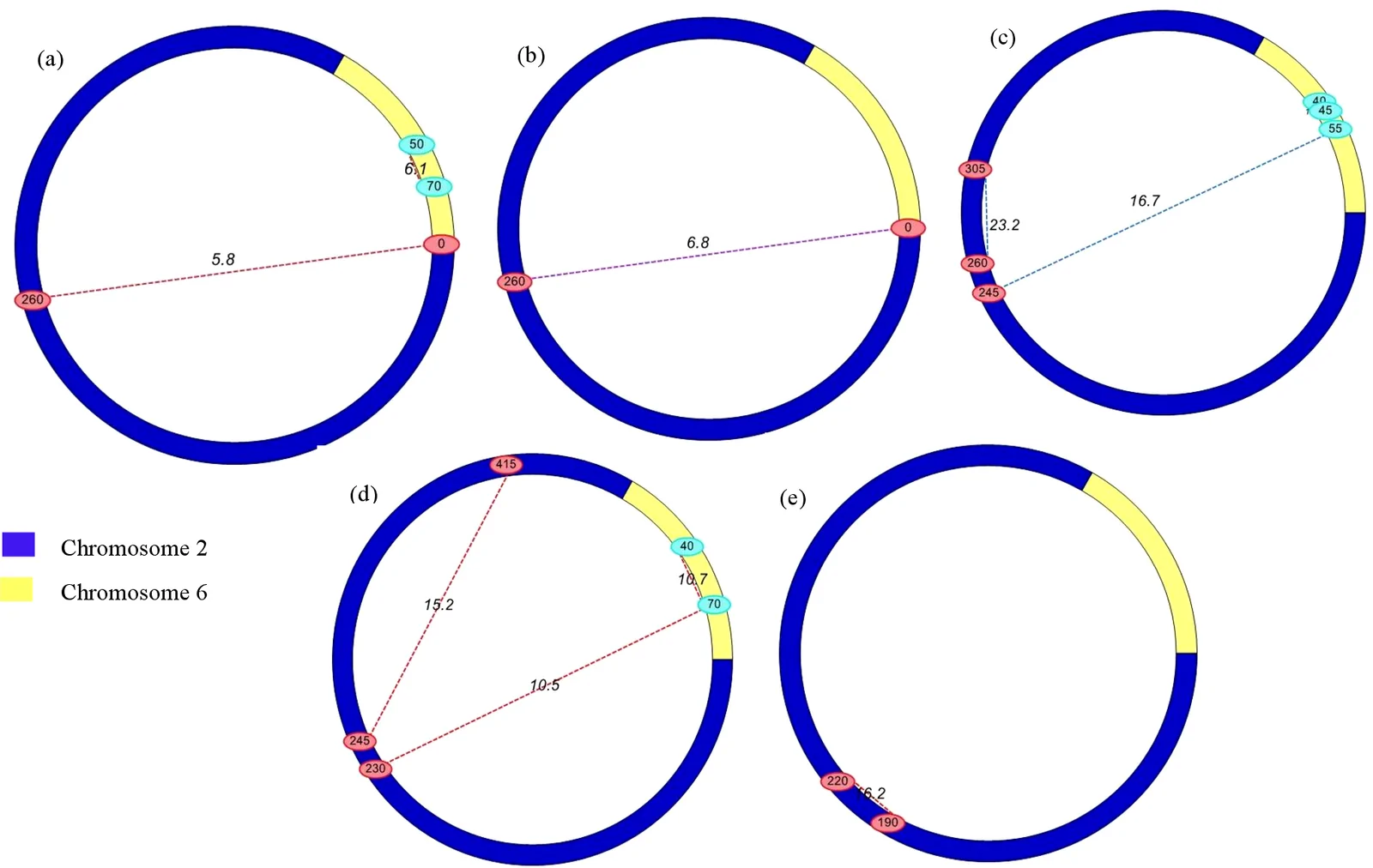
Epistatic QTL interactions for anthracnose fruit rot resistance on chromosomes 2 and 6: **(a)** lesion length, **(b)** lesion width, **(c)** lesion area, **(d)** percent lesion area, and **(e)** disease score.

Bayesian epistatic QTL mapping was performed using the R/qtlbim framework, which identified five significant digenic interactions for DS, based on high *R*² values and low *p*-values. The strongest epistasis was observed between CABQ2.134 and CABQ2.387 (*R*² = 0.175, RMSE = 2.42, *p* = 0.000063). Additional interactions included CABQ2.387 × CABQ6.76 (*R*² = 0.102, *p* = 0.0479), an inter-chromosomal interaction, and CABQ2.10 × CABQ2.387 (*R*² = 0.146, *p* = 0.000701), CABQ2.134 × CABQ2.341 (*R*² = 0.114), and CABQ2.387 × CABQ2.100 (*R*² = 0.109), which represent intra-chromosomal interactions. These results corroborate the epistatic hotspots identified through ICIM-EPI, particularly emphasizing CABQ2.387 as a major hotspot involved in multilocus control of anthracnose fruit rot resistance (**Supplementary Table 9**; **Supplementary Figure 5**).

### Validation of significant QTLs in the IHR 4491 × PBC 80 population

To validate QTLs identified in the IHR4491 × PBC81 F_2_ population, we extended these markers to another F_2_ population of IH4491 × PBC80; ICIM-ADD analysis was conducted using genotypic data and phenotypic data on disease resistance-related lesion traits against *C. truncatum* isolate IIHR-Ct1. QTLs associated with LA, PLA, and DS were consistently detected within the CABQ2.172–CABQ2.157 interval on chromosome 2. The LA QTL at 152 cM showed a LOD of 3.55 and a PVE of 1.12%, with both additive (–1.58) and dominance (–1.98) effects contributing to resistance. The PLA QTL at 149 cM (LOD = 4.67, PVE = 1.27%) showed a minor positive additive effect (2.15) and a strong negative dominance effect (–4.70), suggesting heterozygote advantage. For disease score, a QTL at 137 cM (LOD = 3.11, PVE = 0.87%) showed negligible additive (0.067) and notable negative dominance (–2.50) effects. Based on marker positions, this validated chromosome 2 QTL was designated as *AnR-PBC80-C2*. These results confirm the stability of the QTL region and highlight the role of non-additive gene action in resistance (**Supplementary Table 10**; **Supplementary Figure 6**). However, the *AnR-PBC81-C6* QTL on chromosome 6 could not be validated, indicating complex genetics in the PBC 80-based F_2_ population.

### Candidate genes in refined QTL regions on chromosomes 2 and 6

Refined QTL mapping for anthracnose fruit rot resistance identified a major QTL region on chromosome 2 (836.18 kb; 133.64–134.47 Mb) and a minor-effect QTL region on chromosome 6 (135.15 kb; 249.08 - 249.21 Mb), which are epistatic in nature. Within these regions, 17 potential putative candidate genes were annotated (https://solgenomics.net/) based on function and role in plant defense mechanisms. The chromosome 2 region contained 14 genes, including 6 encoding FAD-binding PCMH-type domain proteins, and others related to vesicle trafficking, transcriptional regulation, DNA processing, and RNA metabolism. Notably, *Cbp02g10520.1*, encoding *Accelerated Cell Death 11* (*ACD11*), was identified near 134.45 Mb, which is a known regulator of hypersensitive response and cell death, supporting its candidacy in resistance. On chromosome 6, three putative candidate genes—a bZIP transcription factor (*Cbp06g29320.1*), an F-box/LRR protein (*Cbp06g29330.1*), and a nuclear phosphoprotein (*Cbp06g29380.1*)—were linked to stress signaling and pathogen recognition (**Supplementary Table 11**).

### Consensus map and meta-analysis of QTLs

A total of 119 QTLs, including QTLs identified in the present study, distributed across all 12 chromosomes, were used for MQTL analysis to identify consensus genomic regions and assess their colocalization with the QTLs detected in this study. The majority of the compiled QTLs were associated with resistance to diseases caused by *Phytophthora capsici* (55.65%) and *Colletotrichum* spp. (27.83%) (**Supplementary Figure 7A**). The reported QTLs had LOD scores ranging from 2.26 to 33.00, while the PVE varied between 0.55% and 70.38% (**Supplementary Figure 7B**). A high-density consensus genetic map comprising 23,596 SNP markers, with an average density of 0.084 cM per SNP, was constructed and utilized for MQTL analysis (**Figure 4**). Following the meta-analysis, 105 QTLs were projected into 36 MQTLs across all chromosomes (**Figure 5**), while 13 QTLs remained as singletons. Among them, MQTL 5.4 showed the highest LOD score and PVE, whereas MQTL 10.2 had the lowest LOD score and MQTL 12.1 exhibited the lowest PVE (**Supplementary Table 12**). MQTL 5.3 contained the highest number of projected QTLs (*n* = 24) (**Supplementary Figure 7C**). Anthracnose fruit rot resistance QTLs on chromosome 2, including two previously reported QTLs (RA932g and RA932r) reported by Mahasuk et al. (2016) and three trait-specific QTLs identified in the present study (*qAfrLL2.1*, *qAfrLA2.1*, and *qAfrLA2.2*; within major QTL *AnR-PBC81-C2*), were colocalized within meta-QTL MQTL2.2, indicating that this genomic region is conserved. The QTL (*AnR-PBC81-C6;* qAfr6.1) on chromosome 6 remained as a singleton and did not colocalize with any previously reported QTLs. This QTL represents a potentially novel genomic region associated with anthracnose fruit rot resistance. MQTL2.1 colocalized with QTL (E1-My-2) for *Phytophthora* root rot caused by *P. capsici* resistance previously reported by Siddique et al. (2019) and is separated from MQTL2.2 by approximately 1 cM. The close physical proximity of these MQTLs highlights this region as a potential hotspot for fungal disease resistance.

**Figure 4:**
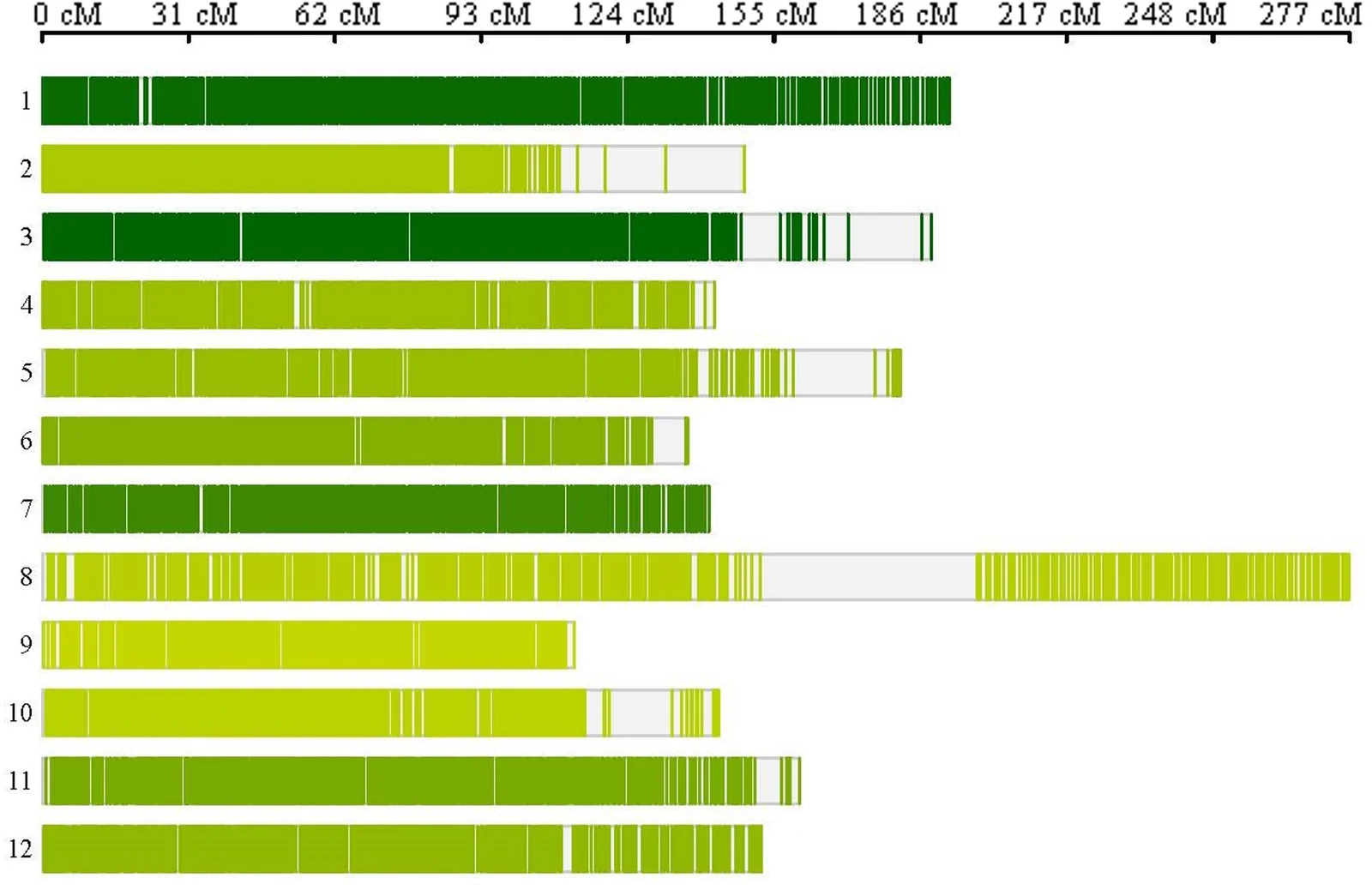
SNP distribution of the high-density consensus map of used for MQTL analysis.

**Figure 5:**
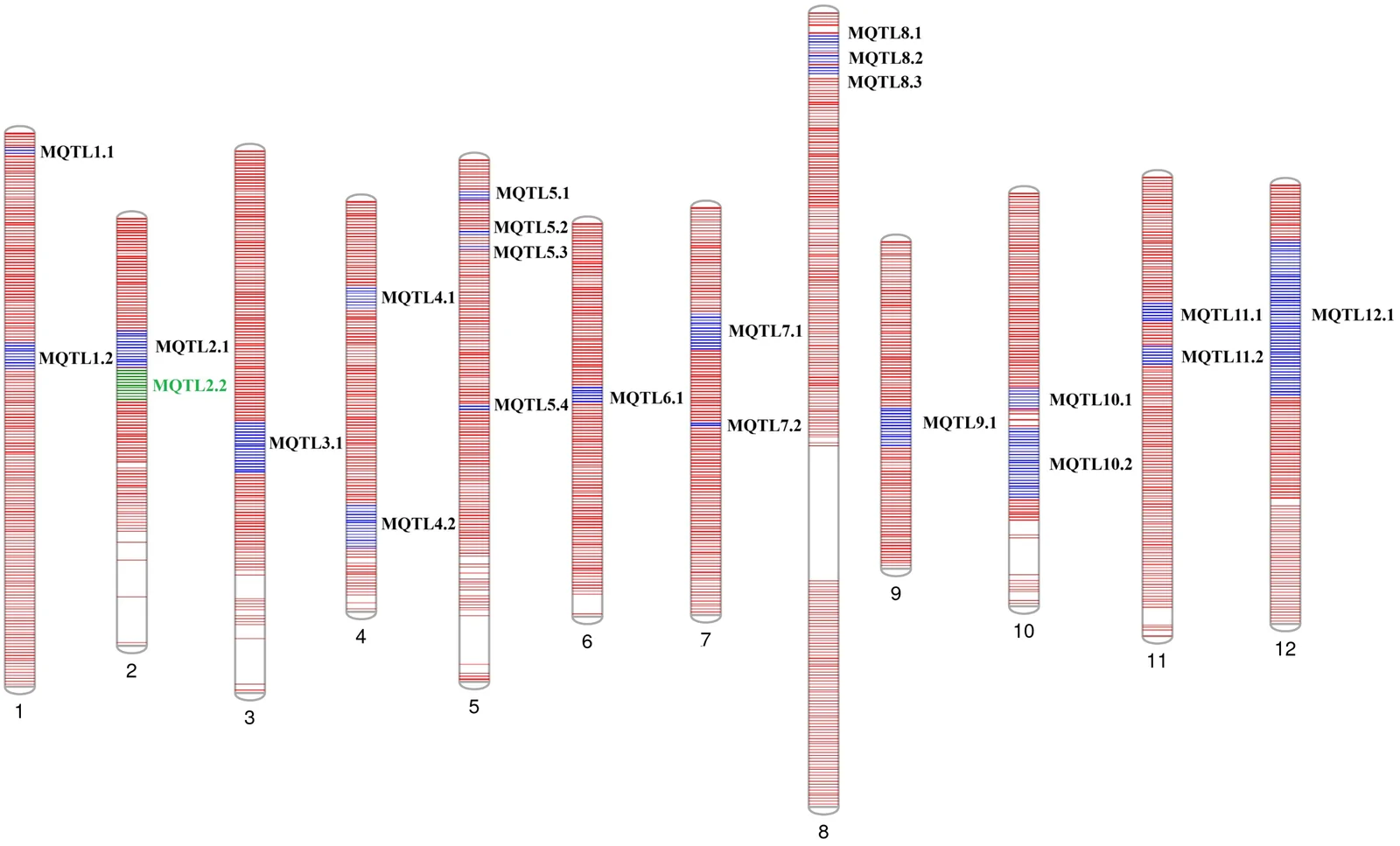
Chromosomal distribution of meta-QTLs (MQTLs) associated with disease resistance in *Capsicum* spp. across the 12 chromosomes. Red lines represent markers constituting the consensus linkage map compiled from published studies, whereas blue lines indicate the consensus MQTL regions identified through meta-analysis. MQTL names are shown adjacent to their corresponding genomic positions. The green-highlighted MQTL2.2 denotes the region colocalized with the QTL identified for anthracnose fruit rot resistance in the present study.

## Discussion

Anthracnose fruit rot, caused by *C. truncatum*, is a major biotic stress affecting chili production and causing severe losses in fruit quality. Chemical control during the fruit ripening stage poses concerns regarding pesticide residues, thereby affecting export value. Consequently, host plant resistance and the development of resistant cultivars remain the most sustainable and economically viable management strategies. However, molecular mapping studies for anthracnose resistance in chili are limited, and most cultivated *C. annuum* accessions are highly susceptible. Resistant sources have been predominantly identified in *C. baccatum* (PBC80, PBC81, and PI594137) and *C. chinense* (PBC932) (Gniffke et al., 2013). The utilization of *C. baccatum* resistance in breeding programs is constrained by cross-incompatibility with *C. annuum*, which remains a major impediment to practical resistance breeding.

Previous inheritance studies have reported diverse genetic models for anthracnose fruit rot resistance, including monogenic recessive inheritance (Mahasuk et al., 2009), duplicate gene action (Lin et al., 2007), and polygenic inheritance controlled by multiple QTLs (Lee et al., 2010). Earlier breeding efforts employed a tri-species bridge-cross method (*C*. *annuum* × *C*. *chinense*) × *C. baccatum* PBC80 to facilitate the introgression of anthracnose fruit rot resistance into *C*. *annuum* (Yoon and Park, 2005). Suwor et al. (2017) introgressed resistance to anthracnose fruit rot caused by *C. acutatum* Ca153 from PBC80 into a *C. annuum* background. They employed embryo rescue coupled with MAS using SCAR-InDel and SSR-HpmsE032 markers. QTLs on LG2, LG4, LG9, and LG12 associated with resistance have been identified in two populations, i.e., *C. annuum* Bangchang × *C. chinense* PBC932, and *C. baccatum* PBC80 × *C. annuum* CA1316 against *C. truncatum* isolate “158ci” and *C. scovillei* pathotypes PCa2 and PCa3 (Mahasuk et al., 2016). Several other studies identified QTLs on chromosomes 3, 5, 7, and 10 in *C. annuum* and *C. chinense* (Sun et al., 2015; Wang, 2011; Kim et al., 2010). Recently, through classical GWAS in 197 C*. chinense* accessions, using 53,518 SNPs and phenotypic data of disease score, SNPs on chromosomes 5, 7, 10, and 12 were identified to be associated with resistance against *C. acutatum* KSCa-1 (Ro et al., 2023). These studies collectively indicate that resistance is highly dependent on the host species, pathogen isolate, and genetic background. Therefore, identifying QTLs associated with resistance to prevalent, region-specific virulent isolates is critical for developing reliable markers and accelerating marker-assisted resistance breeding (Foolad and Panthee, 2012). In the present study, genetic analysis in the F₂ population of PBC 81 × IHR 4491 cross showed that chi-square analysis fit a 13:3 segregation ratio, indicating inhibitory gene action. GBS-based GWAS marker–trait association analysis identified 19 significant QTNs, with five major SNPs on chromosome 2 and seven on chromosome 6, confirming the involvement of two major genomic regions along with several minor loci in resistance to *C. truncatum* isolate IIHR Ct-1. Although a GWAS-like marker–trait association approach was employed in the present study, the use of a biparental F₂ population may limit mapping resolution compared to classical GWAS conducted in diverse natural populations due to extended LD (~2 Mb) and limited allelic diversity. Nevertheless, this approach enabled genome-wide identification of resistance-associated loci, which were subsequently refined through targeted QTL mapping and SSR marker validation. Unlike earlier studies that reported QTLs on chromosomes 3, 5, 7, and 10 in *C. annuum* and *C. chinense* (Sun et al., 2015; Wang, 2011; Kim et al., 2010), our results suggest distinct resistance loci in *C. baccatum*, likely influenced by species-specific interactions with *Colletotrichum* and population structure (Mahasuk et al., 2009; Siddique et al., 2019). Classical QTL mapping using SSR markers refined the resistance loci to 836.18 kb on chromosome 2 and 135.15 kb on chromosome 6, flanked by tightly linked markers. The relatively low phenotypic variance explained by individual QTLs is attributable to the epistatic nature of resistance, which was further supported by ICIM-EPI and Bayesian epistatic QTL mapping. Significant inter- and intra-chromosomal interactions, particularly between chromosomes 2 and 6, revealed that epistasis contributes substantially to resistance expression, consistent with previous reports on complex disease resistance traits (Mahasuk et al., 2016; González et al., 2015; Lozada et al., 2021; Torgeman and Zamir, 2023). The SSR markers flanking these refined QTL regions represent valuable tools for marker-assisted introgression of resistance into elite genetic backgrounds.

Annotation of the refined QTL intervals revealed 17 putative candidate genes associated with defense-related processes, including redox regulation, vesicle trafficking, transcriptional regulation, and stress signaling. On chromosome 2, notable genes include *Cbp02g10390.1*, *Cbp02g10400.1*, and *Cbp02g10420.1*, encoding FAD-binding proteins involved in redox reactions and potentially contributing to reactive oxygen species (ROS)-mediated defense responses during pathogen invasion (Torres et al., 2006). The region also harbored *Cbp02g10440.1*, a Brefeldin A-inhibited guanine nucleotide-exchange protein implicated in vesicle trafficking, which may facilitate transport of defense-related proteins and antimicrobial compounds to infection sites (Yun and Kwon, 2017). *Cbp02g10520.1* encoding ACD11 was identified near 134.45 Mb and is a known regulator of hypersensitive response and programmed cell death, supporting its candidacy in anthracnose resistance (Brodersen et al., 2002; Fukuoka et al., 2009). On chromosome 6, key genes include *Cbp06g29320.1* (BZIP transcription factor), which may regulate salicylic acid- and jasmonic acid-mediated defense signaling and pathogen-responsive gene expression (Alves et al., 2013); *Cbp06g29330.1*, an F-box/LRR-repeat protein involved in pathogen recognition and immune signaling (Lee et al., 2020; Xu et al., 2009); and *Cbp06g29340.1*, associated with ROS detoxification and oxidative stress responses. These candidate genes represent promising targets for functional validation and marker-assisted breeding to enhance anthracnose resistance in chili (Zhong et al., 2021; Kim et al., 2017).

QTL mapping has facilitated the identification of genomic regions conferring disease resistance; however, reported QTLs often vary widely across genetic backgrounds, environments, and marker systems. MQTL analysis integrates QTL information from multiple studies to identify stable consensus regions and resistance hotspots, thereby improving reliability for breeding and candidate gene discovery (Goyal et al., 2024; Saini et al., 2022). In the present study, MQTL analysis was employed to integrate QTL information associated with resistance to anthracnose fruit rot, *Phytophthora* root rot and *Phytophthora* leaf blight, bacterial wilt, bacterial leaf spot, and CMV. MQTL2.2 harbored trait-specific QTLs (*qAfrLL2.1*, *qAfrLA2.1*, and *qAfrLA2.2*; within major QTL *AnR-PBC81-C2*) identified in the present study and previously reported QTLs (Mahasuk et al., 2016). Although Mahasuk et al. (2016) used both *C. annuum* × *C. chinense* and *C. baccatum* × *C. annuum* populations, the QTLs colocalizing with MQTL2.2 were mapped specifically in the *C. annuum* × *C. chinense* population. The genomic interval encompassing MQTL2.1 and MQTL2.2 thus represents a QTL hotspot for resistance to multiple fungal diseases (Rahmanzadeh et al., 2022; Barmukh et al., 2022). In contrast, *AnR-PBC81-C6* QTL did not colocalize with any previously reported QTLs or MQTL regions, suggesting that it may represent a putative novel resistance locus with potential utility in marker-assisted breeding.

## Conclusion

This study provides a comprehensive molecular and genetic dissection of anthracnose fruit rot resistance caused by *C. truncatum* isolate IIHR Ct-1 in *C. baccatum*, including the identification and refinement of major additive and epistatic QTLs on chromosomes 2 and 6. A major QTL region on chromosome 2 (836.18 kb; 133.64–134.47 Mb; *AnR-PBC81-C2*) and a minor-effect QTL region on chromosome 6 (135.15 kb; 249.08 - 249.21 Mb; *AnR-PBC81-C6*), exhibiting epistatic interactions, were identified. The integration of GBS-based SNP discovery, SSR marker development in targeted QTL regions, and high-resolution QTL mapping enabled the identification of tightly linked markers. Within these regions, 17 putative candidate genes were annotated based on function and role in plant defense mechanisms, which represent promising candidate genes. Flanking and peak markers for QTLs on chromosome 2, i.e., CABQ2.62, CABQ2.172, and CABQ2.341, and on chromosome 6, i.e., CABQ6.2, CABQ6.27, and CABQ6.78, can be explored in marker-assisted breeding to accelerate the resistance introgression into different genetic backgrounds. MQTL analysis further established MQTL2.2 as a stable resistance hotspot conserved across genetic backgrounds, while the *AnR-PBC81-*C6 on chromosome 6 locus was found to be a putative novel resistance region. These findings provide valuable genomic resources and validated markers that can facilitate marker-assisted introgression of anthracnose fruit rot resistance loci into elite backgrounds for durable resistance.

## Data Availability

The original contributions presented in the study are included in the article/**Supplementary Material**. Sequencing data have been deposited in the NCBI Sequence Read Archive under BioProject accession PRJNA1391877. Further inquiries can be directed to the corresponding authors.
