# Supplementary material for: QTL mapping and identification of linked markers for anthracnose fruit rot resistance in Capsicum baccatum L
Source: Front Plant Sci. 2026 Sep 23;17:1809315. doi: 10.3389/fpls.2026.1809315 (PMC13642782; doi:10.3389/fpls.2026.1809315)
Supplement: Supplementary file 1 [file DataSheet1.doc]

Supplementary Fig.1 Consensus Linkage Map of SNP Markers across 12 Chromosomes of *C. baccatum*

Supplementary Fig. 2 Genome-wide linkage disequilibrium (LD) decay based on GBS-derived SNP markers in C.*baccatum*

(a)

(b)

Supplementary Fig. 3. Locus specific linkage Map of IHR4491× PBC81 on (a) Chromosome 2 & (b) Chromosome 6

**CABQ2.10**

**CABQ2.11**

**CABQ2.156**

**CABQ2.18**

**CABQ2.60**

**CABQ2.347**

**CABQ2.106**

**CABQ2.118**

**M**

**100bp**

(a)

**P1	P2**

**100bp**

**M**

**1	2	3	4	5	6	7	8	9	10	11	12	13	14	15	16	17	18	19	20	21	22	23	24	25	26**

(b)

Supplementary Fig. S4. SSR marker polymorphism analysis in the IHR4491 × PBC81 population: (a) polymorphic SSR markers between the parental lines PBC81 and IHR4491; (b) gel amplification profile of SSR marker CABQ2.62 in the IHR4491 × PBC81 F₂ population. M: 100bp ladder; P1, PBC81; P2, IHR4491; lanes 1–26 represent individual F₂ plants.

Supplementary Fig. 5. Detection of Significant Epistatic Interactions between SSR Markers using R studio software (Yandell *et al*., 2007)

Lesion Area

Lesion length

Lesion width

Percent Lesion Area

Disease score

Supplementary Fig. 6. QTL-LOD Profiles for Anthracnose fruit rot resistance on chromosomes 2 in IHR 4491 (S) x PBC 80 (R) F_2_ population

Supplementary Figure 7. Disease resistance QTLs utilized for MQTL analysis in *Capsicum* spp. (a) Proportion of disease resistance QTLs in *Capsicum* spp. utilized for MQTL analysis. (b) Distribution of LOD scores and phenotypic variance explained (PVE) of QTLs used in the MQTL analysis. (c) Chromosomal distribution of projected QTLs identified by MQTL analysis in *Capsicum* spp.
